# Changes in female football players’ in-season training load, intensity and physical performance: training progression matters more than accumulated load

**DOI:** 10.3389/fspor.2024.1454519

**Published:** 2024-10-24

**Authors:** Eero H. J. Savolainen, Johanna K. Ihalainen, Tomi Vänttinen, Simon Walker

**Affiliations:** ^1^ Faculty of Sport and Health Sciences, University of Jyväskylä, Jyväskylä, Finland; ^2^ Finnish Institute of High Performance Sport KIHU, Jyväskylä, Finland; ^3^ Neuromuscular Research Center, University of Jyväskylä, Jyväskylä, Finland

**Keywords:** soccer, women, periodization, fitness, monitoring

## Abstract

**Introduction:**

This observational study investigated: (1) potential changes in female football players’ in-season training load, intensity and physical performance, and (2) if in-season accumulated training load, intensity, or their progression are associated to changes in physical performance.

**Methods:**

Thirty-five national level female players (∼21 years, *n* = 35) from three top-teams of the Finnish national league participated. Players performed tests at the beginning and at the end of the 27-week in-season. Tests were: 30-m sprint, countermovement jump (CMJ) and 1,200-m shuttle run, used to calculate maximal aerobic speed (MAS). Players’ external and internal training load and intensity were monitored in all on-field training sessions and official matches (3,941 data samples) using Polar Team Pro system.

**Results:**

Training load decreased towards the end of the in-season (*p* < 0.05), but intensity remained stable. No changes in physical performance test results occurred from before to after in-season tests at a group level. Change of CMJ correlated negatively with accumulated training load, intensity and progression of total distance (TD) and low-intensity running distance (LIRD) (*r* = −0.398 to −0.599, *p* < 0.05). Instead, development of MAS correlated positively with progression of TD and LIRD intensities (*r* = 0.594 and 0.503, *p* < 0.05). Development of both CMJ and MAS correlated positively with intensity progression of very-high-intensity running distance (VHIRD) and number of accelerations and decelerations (*r* = 0.454–0.588, *p* < 0.05).

**Discussion:**

Reduced training load over the in-season is not detrimental for players’ physical performance when training intensity progressively increases. Intensity progression of VHIRD, moderate- and high-intensity accelerations and decelerations are indicators of both MAS and CMJ development, respectively.

## 1 Introduction

Physical performance plays a major role for modern national and international level female football players, since studies have shown that higher-level players perform better in speed (1), strength or power (1–3) and endurance (2–4) tests compared to lower-level players. The differences observed in the tests are meaningful because performance in the selected physical performance tests is associated with high-intensity running during matches (5), and the amount of high-intensity running is a determining factor between competition levels (6).

Previous studies have shown that female players’ physical performance remains stable (7–10) or decreases (11) in endurance tests, remains stable in strength and power tests of the lower limbs (7–10), and remains stable (10) or decreases (7) in speed tests during the in-season. These findings are logical, as the football in-season lasts several months and players need to perform at a high-level week after week to achieve the best outcome in the league (12). Thus, in team sports, the aim is typically to maximize physical performance in the pre-season and then try to maintain that level of physical performance throughout the in-season (13). A similar trend has been found in studies that have quantified female football player's training load (i.e., accumulated absolute value) or intensity (7, 14) (i.e., accumulated values relative to duration) (15). In general, training load is highest during pre-season (7). During the in-season, load and intensity have shown to be highest at the beginning and decreasing towards the end of the in-season (7, 14).

However, it is possible to develop football players’ performance also during the in-season, as has been shown with male players (16–18). These studies have shown positive and negative associations between accumulated training load (accumulated absolute training load in selected metric during observation period e.g., accumulated total distance covered) and development in physical performance tests in 6–9-week periods during the in-season (16–18). There is very limited evidence of such a dose-response relationship (the magnitude of a physiological response, depending on the exposure to a given training stimulus after a certain period) in female players. Goncalves et al. (10) investigated the associations between accumulated training load, measured by session rate of perceived exertion (sRPE), and development in several physical performance tests over 4 weeks of pre-season and 18 weeks of the in-season and found no associations. The team dose-response approach may be problematic as the training stimulus needed for adaptation is dependent on individual characteristics such as training status, nutrition or genetics (19). For example, Mara et al. (7) showed that female football players’ performance in the Yo-Yo intermittent recovery test level 2 at the beginning of the pre-season was associated to the pre-season's accumulated external training load, suggesting that players with higher training status can generate/tolerate a higher external load over the subsequent weeks. Therefore, it seems plausible that players with a better physical performance level may need a greater stimulus to improve. Thus, the approach of previous studies that do not consider baseline training status is weakened when aiming to associate accumulated training load with performance development throughout the season. Typically, this approach does not include the potential effect of training progression (increased training load or intensity over time), even though load progression is one of the key principles of physical training, i.e., an important factor to optimize training adaptations (20). Furthermore, previous studies for male and female football players have only investigated the associations between development and accumulated training load (10, 16–18), while the role of intensity on physical performance development has not been studied, although generally training adaptations are dependent on training intensity (21). To the authors’ knowledge, there are no previous studies that have investigated the associations between football training intensity or progression of training and performance development, even though they are general principles of physical training.

Consequently, the aims of this study were to investigate: (1) potential changes in female football players’ in-season training load, training intensity and physical performance and (2) whether in-season accumulated training load or intensity, or progression of these factors is associated to changes in physical performance. Based on studies conducted with a single team, we hypothesized that: (1) training load and intensity would decrease towards the end of the in-season (7, 14) and there would be no changes or performance decrease in physical performance tests between before and after in-season tests (7–11) and (2) accumulated training load or intensity would not be associated with changes in physical performance test results (10).

## 2 Materials and methods

### 2.1 Study design

**Figure 1:**
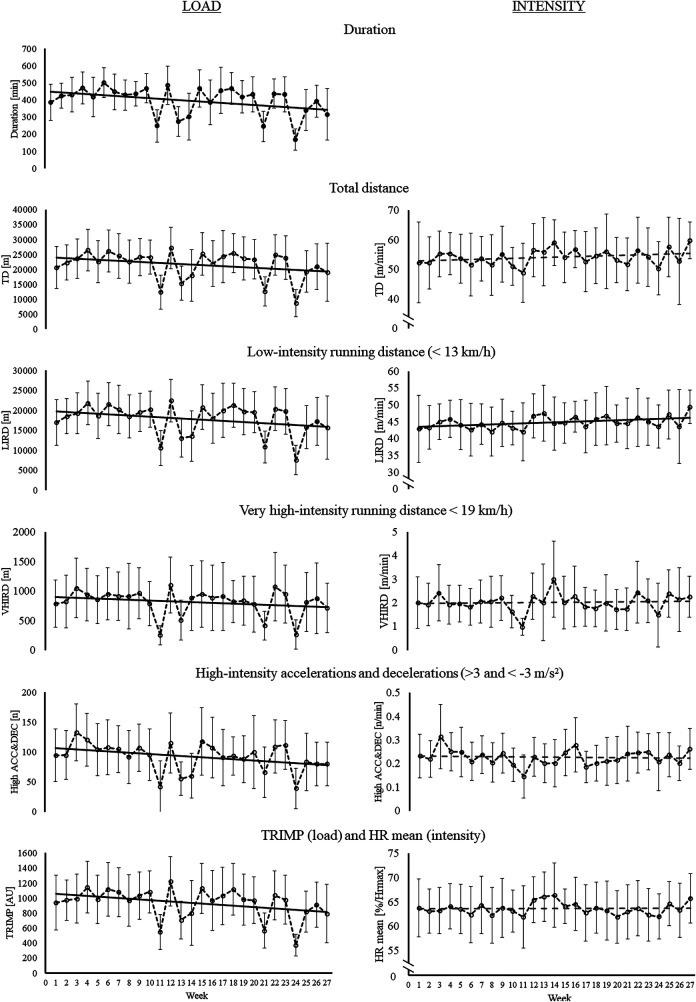
Changes in the key weekly training load (left panel) and average intensity (right panel) variables over the in-season. Black solid linear trend line represents a significant (*p* < 0.05) change over the in-season and gray dashed line represents non-significant (*p* > 0.05) change.

In this observational study, internal and external training load and intensity of players from three top-teams (finished in the top 4 positions in the league) of the Finnish national league were observed over the 27-week in-season 2023, in which the teams played 23 league matches and 1–4 cup matches. Players’ physical performance was measured by field tests at the beginning (April, weeks −3 or −2) and at the end the in-season (October, weeks 26 or 28). These three teams were selected because their competitive success was relatively similar, all teams used the same training load monitoring system, and including three different teams rather than just one, as done in similar previous studies with female (7, 10, 14) and male (16–18) players, would allow better generalizability of the results. There were three international match windows during the data collection period (weeks 5, 13–14 and 24, Figure 1) and national team selected players’ (*n* = 10) data from national team camps was not possible to include in the research. However, these players were included to the study because they still fulfilled the study's inclusion criteria.

### 2.2 Participants

Sixty-six national level (22) female football players volunteered to participate. Thirty-five players (21.1 ± 2.8 years, 166 ± 5 cm, 64 ± 5 kg. *N* = 13 from team A, *n* = 10 from team B and *n* = 12 from team C) met the inclusion criteria: (1) outfield position (8 goalkeepers were excluded), (2) played the full season in the team (7 players who moved to other clubs for loan or permanently were excluded), (3) participated in at least 80% of their teams’ training sessions or matches during the in-season (16 players who participated <80% team's sessions due to injuries, illnesses or other reasons or were not selected to match squad were excluded) (23) and (4) participated to initial physical performance tests (9 players who did not voluntarily participate to tests were excluded). All participants who fulfilled the inclusion criteria did not participate to all post-tests, due to injuries, illnesses or concurrent national team camp and, thus, the sample size in pre- and post-in-season comparisons is 21–26 depending on the test.

Players and parents of players younger than 18 years were informed, verbally and in written form, of possible risks and discomforts associated with the study procedures and had the opportunity to discuss the study with the researchers. Players then signed a consent form. The study was approved by the Ethics Committee of the university (1,375/13.00.04.00/2022) and conducted according to the Declaration of Helsinki (2013), except for registration in a database.

### 2.3 Study procedures

#### 2.3.1 Training load and intensity monitoring

During the observation period, 3,941 samples from football training sessions, league and cup matches were collected by Polar Team Pro player tracking system (Polar Electro Oy, Kempele, Finland) with Global Positioning System (GPS) sampled at 10 Hz and HR monitoring. Players performed systematic strength training based on their teams’ fitness coach's programming throughout the in-season, but this training was not taken into account or monitored in the present study. Good-to-moderate reliability [<5% coefficient of variation (CV)] and validity for total distance, linear running and team-sport simulation circuit have been reported for the system (24). Seventeen training load samples were excluded due to issues (e.g., top speed >35 km/h) in data collection. All variables are referred to as load (i.e., absolute value e.g., meters) and intensity (i.e., value relative to duration e.g., meters per minute) as suggested by Gaudino et al. (15) The following variables were used to represent external training load and intensity: total duration [min], total distance (TD) [m and m/min], distance covered in low-intensity running (LIRD, <13 km/h) [m and m/min], distance covered in high-intensity running (HIRD, 13–19 km/h) [m and m/min] distance covered in very-high-intensity running (>19 km/h) [m and m/min], (5) number of low- (1–2 and −1 to −2 m/s^2^), moderate- (2–3 and −2 to −3 m/s^2^) and high-intensity (>3 and <−3 m/s^2^) accelerations and decelerations [n and n/min] (25). Edward's training impulse (TRIMP) (26) was used to represent internal load and average heart rate to represent internal intensity.

#### 2.3.2 Physical performance tests

Players were tested by 30-m sprint test, countermovement jump and 1,200-m shuttle running test (i.e., Bronco test). Tests were performed in an indoor football field selected by the teams. Before the tests, coaches were instructed to periodize either rest or a light training session the day prior to the tests. The teams’ fitness coaches oversaw the warm-up before the tests. A standardized warm-up protocol was introduced to coaches to follow (included 5 min jogging, activation and mobility exercises such as lunges, squats and single leg deadlifts and three sprints and jumps with increasing intensity), but coaches could modify in-line with their team's habits. The test session started with sprint and jump tests and ended with the 1,200-m shuttle running test. All tests in this study were led by the same researcher.

The 30-m sprint test was performed on artificial turf wearing football shoes. Players began 70 cm behind a photocell gate (Newtest Oy, Finland), which was one meter from the ground. Players performed three trials separated by three minutes rest, and the best time to the nearest 0.01 s was used in analyses. CV between three trials was 0.9 ± 0.8% for sprint time, which is lower than the ∼3.9% reported previously with female football players (27).

The CMJ test was performed hands on hips on a hard surface wearing running shoes. Jump height was determined from the jump's flight time to the nearest 0.01 s by using an infrared mat (Custom built, University of Jyväskylä). Players performed three trials separated by three minutes, and the highest jump was used in analyses. CV between three trials was 2.7 ± 1.4% for CMJ height, which is also slightly lower than previously reported ∼3.9% with female football players (27).

The 1,200-m shuttle running test was performed on artificial turf with the entire team performing the test simultaneously. The aim of the test was to complete the shuttle running track with 20-m, 40-m and 60-m shuttles five times as fast as possible. The test protocol is described more specifically by Kelly & Wood's (28). As a warm-up, players were instructed to jog the track once and then run it once at a self-determined speed in which they thought they will start the test. After the warm-up, players had three minutes recovery before beginning the test. For the test, players wore football shoes. From 1,200-m shuttle running test, maximal aerobic speed (MAS) was calculated by the following formula: MAS = 1,200 m/(test time in seconds – 20.3) (29).

### 2.4 Statistical analysis

Statistical analyses were conducted using IBM SPSS Statistics® software (v28.0, IBM Corporation, Armonk, New York, USA). Results are reported as mean ± standard deviation. The significance level was set at *p* < 0.05. Shapiro-Wilk test showed that in-season accumulated training load, training intensity and physical performance data were normally distributed (TD and VHIRD loads after log-transformation). One-way analysis of variance with Bonferroni *post hoc* tests was used to investigate potential differences in training load, intensity and physical performance and their progression/development between the three teams participating in the study. This analysis was performed to determine the appropriateness of combining the three teams for further analyses.

Generalized estimating equations were used to model the changes in weekly training load and intensity over the in-season. For this, an exchangeable correlation structure and linear distribution of the response variable (training load or intensity variable) were assumed. The selected training load or intensity variable was used as a dependent variable and week as a covariate.

Paired samples *t*-test was used to investigate potential changes in physical performance test results between before and after in-season tests. Effect sizes were calculated as Hedges’ *g* and were classified as: 0.2–0.5 small, 0.5–0.8 medium, >0.8 large effects.

Pearson's product moment correlation was used to assess associations between before in-season test physical performance status and accumulated in-season training load or intensity. This was also used to assess associations between in-season accumulated training load, intensity and percentual change in physical performance test result.

Training progression was determined by calculating the linear slope from each player's weekly load and intensity variables over the 27-week in-season period. Spearman rank correlation was used to assess associations between in-season training progression (slope) and percentual changes in physical performance test result. All correlation magnitudes were classified as: <0.3 weak; 0.3–0.7 moderate, >0.7 strong.

## 3 Results

### 3.1 Differences between the teams

There were no differences in any physical performance tests or their development throughout the in-season between the three teams’ results. From training load and intensity variables only accumulated high ACC&DEC load (F = 4.36, *p* = 0.019) and intensity (F = 4.81, *p* = 0.013) demonstrated statistical differences between the three teams. *Post hoc* tests showed that team A reached higher high ACC&DEC load and intensity than team C (2,563 ± 797 n vs. 1,851 ± 460 n and 0.26 ± 0.07 n/min vs. 0.20 ± 0.04 n/min, respectively).

### 3.2 Changes in training load over the in-season

Weekly total training duration and all training load variables decreased towards the end of the season: total duration B = −3.9, *p* < 0.001; TD B = −178.1, *p* < 0.001; LIRD B = −140.5, *p* < 0.001; HIRD B = −31.2, *p* < 0.001; VHIRD B = −7.6, *p* = 0.002; low ACC&DEC B = −23.3, *p* < 0.001; moderate ACC&DEC B = −5.6, *p* < 0.001; high ACC&DEC B = −1.2, *p* < 0.001; TRIMP B = −9.7, *p* < 0.001 (Figure 1 left panel). Weekly LIRD intensity increased towards the end of the season B = 0.1, *p* = 0.002. Other variables’ weekly intensity did not change (*p* > 0.05) over the in-season (Figure 1 right panel).

### 3.3 Changes in physical performance over the in-season

**Figure 2:**
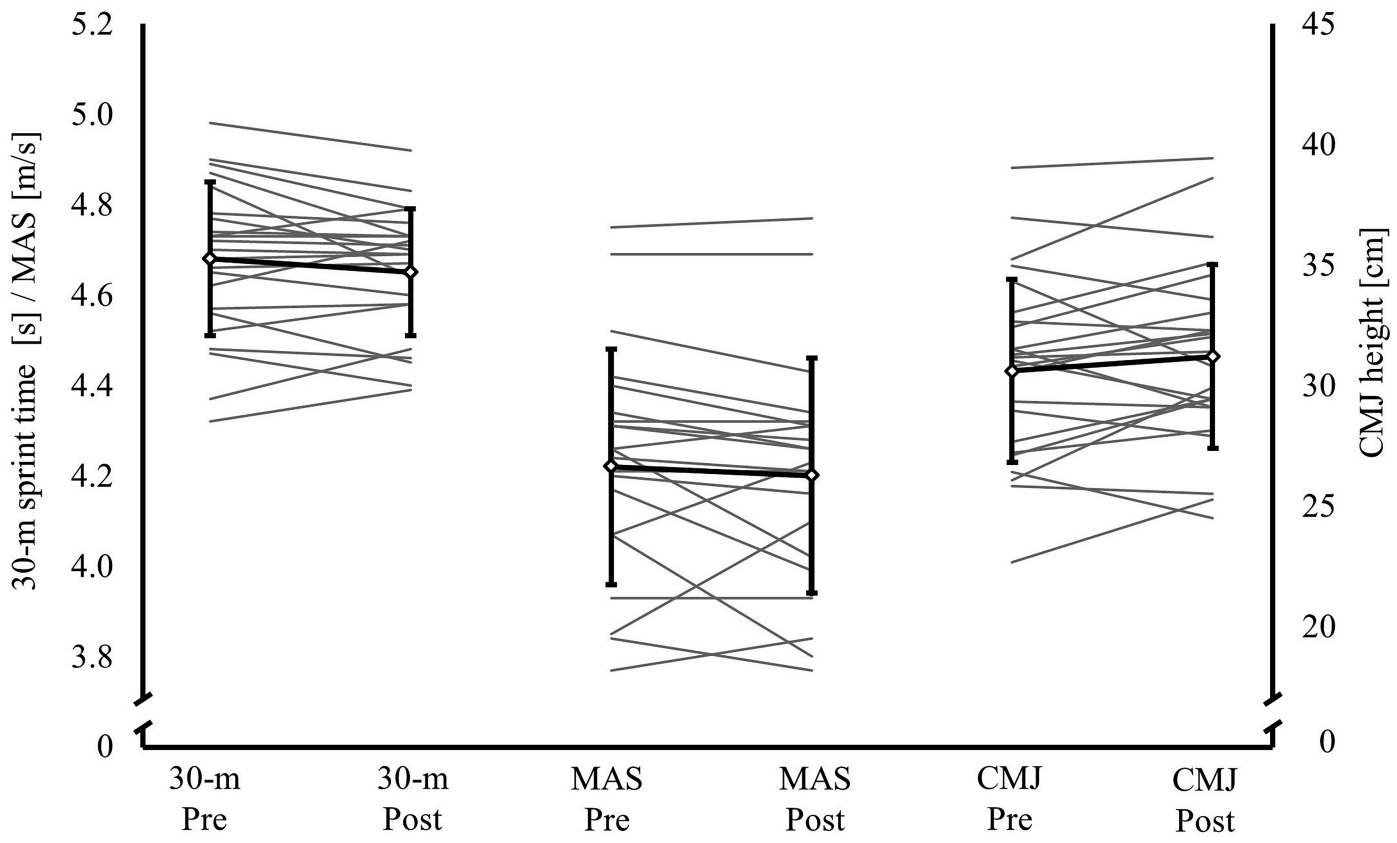
Changes between pre- and post-in-season 30-m sprint time, maximal aerobic speed (MAS) and countermovement jump (CMJ) height values. Black lines represent mean values and SDs and gray lines represent individual players’ changes.

No significant changes in physical performance occurred between before and after in-season tests on a group level as shown in Figure 2. Hedge's *g* values for the group level changes in 30-m sprint time, MAS and CMJ were: *g* = −0.329, *g* = −0.165 and *g* = 0.288, respectively. The range in individual players’ development varied from 4.3% to −2.5% in 30-m sprint time, from 6.1% to −7.1% in MAS and from 12.9% to −11.5% in CMJ height.

### 3.4 Associations between physical performance and in-season accumulated training load and intensity

Several significant, weak-to-strong, correlations were observed between before in-season MAS and 30-m sprint performance, and several in-season accumulated training load and intensity variables (Table 1). However, the only significant correlations between in-season training load or intensity variables and changes in physical performance test results were weak-to-moderate negative correlations between CMJ and accumulated loads and intensities of TD (*r* = −0.398, *p* = 0.046 and *r* = −0.437, *p* = 0.029 respectively) and LIRD (*r* = −0.473, *p* = 0.017 and *r* = −0.599, *p* = 0.002 respectively).

**Table 1:** Correlation coefficients between performance in before in-season physical performance tests and in-season accumulated training load and intensity.

Test		Total duration	TD	LIRD	HIRD	VHIRD	Low ACC&DEC	Mod ACC&DEC	High ACC&DEC	TRIMP (load)/HR mean (intensity)
30-m sprint time (*n* = 35)	Load	0.176	0.022	0.110	−0.056	−0.386*	0.037	−0.121	−0.364*	0.034
	Intensity		−0.176	−0.109	−0.145	−0.311	−0.161	−0.201	−0.420*	−0.099
CMJ height (*n* = 35)	Load	0.074	0.196	0.142	0.196	0.293	0.188	0.193	0.317	0.145
	Intensity		0.239	0.227	0.187	0.053	0.324	0.144	0.257	0.145
MAS (*n* = 35)	Load	0.168	0.361*	0.225	0.596*	0.702*	0.407*	0.563*	0.666*	0.081
	Intensity		0.473*	0.188	0.705*	0.725*	0.541*	0.662*	0.712*	−0.096

TD, total distance; LIRD, low-intensity running distance (<13 km/h); HIRD, high-intensity running distance (13–19 km/h); VHIRD, very-high-intensity running distance (>19 km/h); Low, Mod. and High ACC&DEC, number of low- (1–2 and −1 to −2 m/s^2^), moderate- (2–3 and −2 to −3 m/s^2^) and high-intensity (>3 and <−3 m/s^2^) accelerations and decelerations; TRIMP, Edward's training impulse; HR mean, mean heart rate; CMJ, countermovement jump; MAS, maximal aerobic speed.

*Statistically significant (*p* < 0.05) correlation coefficient between before in-season physical performance test result and in-season accumulated training load or intensity.

### 3.5 Associations between physical performance changes and in-season training load and intensity progression

Significant, weak-to-moderate, positive associations were found between MAS improvement and progression (slope) of TD, LIRD, HIRD, VHIRD, moderate and high ACC&DEC intensities, as well as between MAS improvement and progression of total duration and high ACC&DEC loads. Significant, weak-to-moderate, positive correlations were also found between CMJ improvement and progression of VHIRD, low, moderate and high ACC&DEC intensities. Progression of HIRD, VHIRD, moderate and high ACC&DEC loads also correlated positively with CMJ improvement. Conversely, progression of TD and LIRD intensities were negatively associated with the CMJ improvement, as well as progression of total duration (Table 2).

**Table 2:** Correlation coefficients between progression (slope) of in-season training load and intensity and change (in percentage) from before to after in-season physical performance tests.

Test		Total duration	TD	LIRD	HIRD	VHIRD	Low ACC&DEC	Mod ACC&DEC	High ACC&DEC	TRIMP (load)/HR mean (intensity)
30-m sprint time (*n* = 25)	Load	−0.117	0.077	0.064	−0.016	−0.092	0.010	0.017	−0.134	0.165
	Intensity		−0.296	−0.194	−0.183	−0.399	−0.170	−0.290	−0.267	0.059
CMJ height (*n* = 26)	Load	−0.417*	−0.317	−0.316	0.389*	0.396*	0.383	0.409*	0.499*	0.141
	Intensity		−0.521*	−0.487*	0.381	0.570*	0.484*	0.531*	0.588*	0.201
MAS (*n* = 21)	Load	0.498*	0.396	0.440	0.442	0.411	0.393	0.423	0.511*	0.335
	Intensity		0.594*	0.503*	0.631*	0.454*	0.408	0.551*	0.568*	0.224

TD, total distance; LIRD, low-intensity running distance (<13 km/h); HIRD, high-intensity running distance (13–19 km/h); VHIRD, very-high-intensity running distance (>19 km/h); Low, Mod. and High ACC&DEC, number of low- (1–2 and −1 to −2 m/s^2^), moderate- (2–3 and −2 to −3 m/s^2^) and high-intensity (>3 and <−3 m/s^2^) accelerations and decelerations; TRIMP, Edward's training impulse; HR mean, mean heart rate; CMJ, countermovement jump; MAS, maximal aerobic speed.

*Statistically significant (*p* < 0.05) correlation coefficient between progression of in-season training load or intensity and change from before to after in-season physical performance test result.

## 4 Discussion

This study determined possible changes in training load, training intensity and physical performance of national level female football players during the in-season. Secondly, it determined whether in-season training load, intensity or their progression were associated to changes in physical performance. Also, it aimed to determine if in-season training load, intensity or their progression are associated to changes in physical performance. As hypothesized, female football players’ physical performance did not change over the in-season and training load (including training duration) decreased towards the end of the in-season, on a group-level. However, training intensity remained stable over the season or even increased in LIRD, which indicates that the main factor in decreased training load was decreased training duration. Contrary to our hypotheses, there were significant weak-to-moderate negative correlations between in-season's accumulated training load and intensity of TD and LIRD and development of CMJ height. Further, also progression of these were negatively associated with development of CMJ height. Conversely, progression of TD and LIRD intensity were positively correlated with MAS development. These opposite adaptations from TD and LIRD progression highlight a potential interference effect and challenge coaches to periodize training to achieve desired adaptations. Finally, progression of VHIRD, moderate- high-intensity ACC&DEC intensities correlated positively with both MAS and CMJ development. Therefore, the intensity progression of these mechanically more demanding variables over the in-season appears to be a critical indicator for development of both MAS and CMJ performance, respectively.

At the group-level, none of the physical performance variables changed over the in-season. Generally, all previous studies from USA, Australia and Portugal conducted with a single female team, have shown similar results (7–10). This study combined players from three different teams, which were all top-teams of the Finnish national league. Thus, based on these and previous studies’ findings with a single team, it seems that physical status of female football players remains relatively stable from the start to the end of the in-season, even though there are differences between the league systems (season duration, number of matches, frequency of matches etc.) in different countries. From a coaching perspective, this finding is logical. During the in-season, the main aim is to win the following match and try to maintain players’ physical performance level throughout the in-season (12). Therefore, in-season's training load is typically lower than pre-season (7) and it decreases towards the end of the in-season (7, 14), as shown also in this study in all training load variables (including total duration). In the present study, the intensity remained stable over the in-season or even increased in LIRD. This conflicts with findings from a Norwegian team reported by Karlsson et al. (14) who showed that some training intensity variables also decrease towards the end of the in-season. Therefore, it appears that Finnish teams’ coaches decreased training load mainly by decreasing training duration in this study, while maintaining the intensity at the same level. This type of tapering (decreased load and maintained intensity) has been shown to peak match performance at a mesocycle-level (30). Tapering is an effective strategy to improve maximal power (31), which was also implied in this study where progression of TD and LIRD load and intensity during the in-season was negatively associated to CMJ development.

Furthermore, development in CMJ performance was negatively associated with in-season TD and LIRD loads and intensities, suggesting that a high in-season load or intensity of low-intensity TD/LIRD actions is detrimental for power performance. Similarly, total exposure time (training and matches) and muscular perceived sRPE were negatively correlated to CMJ height over a 9-week in-season period in males (18). These findings agree with tapering theory in that overall training volume should be reduced, and in this case low-intensity volume, but high-intensity actions should be emphasized. In support, progression of in-season's HIRD, VHIRD, moderate- and high-intensity ACC&DEC loads and VHIRD and low-, moderate- and high-intensity ACC&DEC intensities were positively correlated to CMJ development in this study. Thus, the proportion of low- to high-intensity actions may need modification throughout the in-season and more focus on progression of mechanically more demanding variables to improve CMJ performance. One possibility to achieve high VHIRD and ACC&DEC intensities via football-specific exercise could be large-sided games (32). Nevertheless, it should be noted that strength and power training are recommended in addition to football-specific training to maximize power development (33).

In MAS and sprint tests, there were no associations between training load or intensity and changes in physical performance. Similar findings have been shown by Goncalves et al. (10) who observed that female players’ accumulated weekly average sRPE was not associated to changes in their physical performance development. This type of team dose-response relationship approach can be criticized because training status influences what absolute stimulus is required to drive adaptations (19), which was also shown in this study. Weak-to-strong correlations between MAS at the beginning of the in-season and in-season accumulated training load and intensity in several external training load variables were observed. This suggests that players with greater initial aerobic capacity generated higher levels of training load and intensity throughout the season, which has also been previously shown in players from an Australian team (7). Thus, physical performance and in-season's training load and intensities are related, but this does not predict adaptation. To predict adaptations, analyzing methods that account for changes in players’ individual training load and intensity over time are required.

This study showed that progression in training duration and high-intensity ACC&DEC load were positively associated with development of MAS along with progression of TD, LIRD, HIRD, VHIRD, moderate- and high-intensity ACC&DEC intensities. However, excessive training load can increase perceived fatigue (34) and then potentially impair match performance. Therefore, the focus of the in-season's training should be to increase training intensity progressively either by modifying the training formats or by developing players’ physical performance, which should provide a higher training intensity and subsequently improve MAS, as well as possibly increase match running performance (5).

Internal load (TRIMP), intensity (HR average) or progression of these were not associated with either the before in-season test results or physical performance improvement. This finding is surprising considering that the progression of several external load and intensity variables correlated with MAS improvement. Evidence suggests that players train and play at the same internal intensity regardless of their playing standard, but those with higher physical performance are able to generate higher external load (25). Thus, it appears that players whose MAS improved during the in-season were able to progressively generate higher external intensity with similar internal responses in this study. Therefore, monitoring external and internal intensities over the in-season is vital; i.e., if external intensity progressively increases over the in-season and internal responses remain stable, coaches can expect players’ aerobic capacity to be improved without the need for separate testing.

The biggest strength of the present study was that it combined data from three teams from the same league whereas previous studies have typically only observed a single team (7, 10, 14). Therefore, the results draw a more robust picture of the phenomenon than previous studies and indicate a good level of generalizability for national level female football players. However, these are still specific teams, with specific training cultures and, thus, there is a potential bias due to team selection. Also, a novel approach was the investigation on training progression and physical performance development rather than the previous focus on associations between accumulated training load and development. At the same time, it is acknowledged, that the method used to determine training progression in the present study was not sophisticated and, therefore, further research may be needed to optimize the methods.

The biggest limitation of present study was that, as in similar studies, conclusions have been drawn from correlations. Correlation coefficients show only the associations between variables, not causality. Thus, it is possible that players improved physical performance allowing them to generate higher training intensity (i.e., run more VHIRD per minute), which causes positive progression to training intensity (and to load if duration is constant) even though coaches have not planned this in their training prescription. Unfortunately, training load monitoring was limited to football sessions only and players’ training load from strength sessions was not included to the analysis. Therefore, it cannot be confirmed if strength training was associated to improved physical performance and thereafter to higher training intensity in football sessions. Another limitation is the relatively high drop-out rate because of injuries, illnesses and that one team wanted to have the after in-season test during an international match-window when five players were unable to participate. However, sample size has been smaller in previous studies with female players (7–11). The third limitation was that players’ anthropometry or body composition were not reported. Body composition measurement (by bioelectrical impendence) was offered to players, but less than 30% of the players voluntarily participated to the measurement at the beginning of the in-season and, therefore, data was not used. Anthropometry and body composition data would have given valuable information to interpret results since performance in all tests required moving the body's mass. Finally, it is a limitation that the present study focused only on the in-season because pre-season training in Finland is mainly performed indoors due low temperatures in winter. Thus, GPS-data from the pre-season was invalid. However, players’ physical performance was tested at the beginning and at the end of ∼10 weeks pre-season showing significant improvements in 30-m sprint time, CMJ height and MAS (∼2 – 3% depending on the test, *p* < 0.05) over the pre-season. This demonstrates that players’ physical performance was primed (on an individual level) for demands of the in-season, which players were able to maintain (on a group level) throughout the in-season.

## 5 Conclusions

The present study showed that national level female football players’ training load decreased towards the end of the in-season, while training intensity remained stable. As previously observed, players’ physical performance remained stable (on a group level) over the in-season. Only negative associations between the in-season's accumulated training load or intensity and physical performance improvement were observed between TD and LIRD and CMJ. Concurrently, progression of some training load and several training intensity variables positively correlated with CMJ and MAS improvement, which questions the dose-response relationship approach (on a group level) and highlights the role of training progression (on an individual level). Based on the current findings, coaches of national-level female players are advised to focus on training intensity progression in two key ways. Firstly, by enhancing players’ MAS and CMJ performance, which can lead to increased training intensity during football sessions. Secondly, by planning and periodizing football sessions to progressively increase training intensity throughout the season.

## Data Availability

The datasets presented in this article are not readily available because data that support the findings of this study are available from the corresponding author upon reasonable request. Requests to access the datasets should be directed to Eero H.J. Savolainen, eero.h.j.savolainen@jyu.fi.
